# Interpersonal relationships in patients suffering from chronic musculoskeletal pain: a case-control study analyzing core conflictual relationship themes and interpersonal problems

**DOI:** 10.1186/s13030-025-00335-x

**Published:** 2025-07-28

**Authors:** Pernilla Abrahamsson, Bo Vinnars, Annika Lindgren

**Affiliations:** 1 Division of Clinical Psychology, Department of Psychology, Uppsala University, Uppsala, 751 42 Sweden; 2 Department of Clinical Neuroscience, Karolinska Institute, Stockholm, 171 77 Sweden

**Keywords:** Chronic pain, Pain diagnosis, Pain experience, Interpersonal relationships, Interpersonal problems

## Abstract

**Background:**

Psychosocial factors are involved in all types of chronic pain but seem to play a more prominent role in non-specific pain, such as chronic musculoskeletal pain (CMP), compared to a specific pain condition, such as osteoarthritis (OA). We explored if diagnose and the pain experience in patients with CMP predicted more problematic interpersonal relationships compared to patients with OA.

**Methods:**

Nineteen patients with CMP and 16 unmatched clinical controls with OA were measured with the Core Conflictual Relationship Theme coding of clinical interviews (CCRT) and the Inventory of Interpersonal Problems (IIP).

**Results:**

Significant differences in age, work status, and pain experience were found between the groups. Controlling for these variables, components of CCRT were significantly more likely to be disharmonious in patients with CMP compared to patients with OA. Patients with CMP also reported more interpersonal distress in general and socially avoidant-nonassertive problems in particular as their pain experience increased. Conversely, scores of dominant-intrusive behaviours increased as their pain experience decreased. These interaction effects between pain experience and interpersonal problems were not seen in patients with OA.

**Conclusions:**

The impact of interpersonal issues may differ depending on type of pain diagnosis. This study show that interpersonal distress seems to play a more prominent role in non-specific chronic pain compared to a specific pain condition. It is possible that patients whose pain-processing system is burdened by interpersonal problems are more prone to non-specific pain, such as CMP. It could also be that primary pain is a greater challenge to interpersonal relationships. Whether interpersonal distress is a precursor or an additional stressor, it may worsen the condition of primary pain with implications for treatments.

**Supplementary Information:**

The online version contains supplementary material available at 10.1186/s13030-025-00335-x.

Exposure to socially painful situations may play a crucial role in the development and maintenance of chronic pain [1] and the social impact on chronic pain has been increasingly recognized in research [2]. For example, negative responses from significant others have been associated with more pain, poorer physical and mental health, and more fatigue-related symptoms over time [3]. High levels of social isolation predicts much higher levels of pain intensity and disability compared to individuals with low levels of social isolation [4,5]. Conversely, the presence of support and meaningful social ties increase resilience and effective adaption [6]. For example, the impact of pain is reduced when patients perceive others as including and encouraging [7,8], and social support in a work place is a buffer against chronic pain [9]. Bannon et al. [10] found that patients reported physical and mental improvements after an intervention that also addressed their social isolation. From experimental research, it is evident that social connectedness is associated with pain ratings [11,12]. Using fMRI to assess the neural response to the threat of an electric shock, Coan et al. [13] could demonstrate an attenuated threat response if a woman was holding someone’s hand, even if it was a stranger. The effect increased when she was holding the hand of her spouse instead, and even more as a function of marital quality. In a health care setting, a positive relationship between clinician and patient might increase patient’s engagement in treatments, ultimately having an influence on treatment outcomes [14]. However, in patients struggling with interpersonal issues, developing a helpful dialogue with health care providers or any other important person may prove difficult. Support can be present, but not perceived.

In an account of interpersonal pain dynamics, Vervoort and trost [15] highlight the importance of a match between the emotional needs of the person in pain and the caregiving response in order to be helpful. A case-control study by Landa et al. [16] used the core conflictual relationship theme interview to examine interpersonal themes in patients suffering from somatization syndrome according to DSM-IV (including the pain syndrome). Significantly more often than healthy controls did the patients express interactions with others as a rejected desire for closeness, to which they reacted with anger, disappointment, or depression. Whether or how patients made efforts to disclose their wish for closeness remained unclear, but the theory is that expected rejections lead to mistrust and closure in future interpersonal encounters, and the patients scored higher on a measure of mistrust compared to the healthy control [16]. This frustrated need for emotional support is the most common interpersonal theme in psychiatric samples when the core conflictual relationship theme interview is used [17]. With some exceptions of psychosomatic samples (e.g., [16,18]) that interview has not been used with chronic pain patients in medical settings.

When patients with chronic pain are measured with the self-report inventory of interpersonal problems (IIP), they are characterized by being overly nurturant, exploitable, nonassertive, and socially avoidant [19–21]. This is the most common problem in psychiatric samples too [22]. Borys et al. [23] found significantly higher scores of submissiveness in a group of low-back pain patients compared to a healthy control, and they reported higher levels of pain intensity, functional disability, depression, catastrophizing, and health care utilisation compared to patients with lower scores of submissiveness.

Chronic pain includes a large variety of clinical pain conditions [24]. Non-malignant, musculoskeletal pain defined as pain in one or more anatomical regions [25] is an unspecific disorder in the sense that organic findings are out of proportion with the pain intensity and the pain related disability [26]. Although psychosocial factors are involved in all types of chronic pain, they seem to play a stronger role in unspecific pain conditions [27–29]. Biologically, studies indicate that many of the pathways identified as being important for unspecific pain are also important for emotional distress [27]. Patients with unspecific pain report lower quality of life and more emotional distress compared to patients with specific pain conditions (e.g., [30,31]), but research on interpersonal difficulties in patients with chronic musculoskeletal pain (CMP) compared to other pain conditions are scarce. In general, patients are studied within the overarching concept of chronic pain (e.g., [32]). Comparisons are either made within the group (e.g., [5]) or with healthy subjects (e.g., [23]).

A few exceptions should be mentioned. Faucett and Levine [33] found that interpersonal difficulties affected patients with fibromyalgia (an unspecific pain condition) differently compared to patients with arthritis. Hornemann et al. [34] found that patients with non-specific pain disorders, including CMP, reported worse social functioning compared to patients with specific pain conditions, and Telesca et al. [35] found impaired social cognition abilities in patients with non-specific pain compared to patients with specific pain. Finally, McInnis et al. [36] found that women with fibromyalgia or chronic fatigue syndrome reported more secrecy around their illness, which was not found in women with rheumatoid arthritis, osteoarthritis, or multiple sclerosis. With these exceptions, studies of differences in interpersonal factors depending on type of pain diagnose are rare.

This study will explore if diagnose and pain experience matters to interpersonal relationships, comparing two different chronic pain conditions, each representing unspecific and specific pain. Interpersonal relationships were analyzed using the Core Conflictual Relationship Theme interview (CCRT) and the self-report of Inventory of Interpersonal Problems (IIP). It was explored if there is (1) a main effect of diagnose - non-specific musculoskeletal pain (CMP) and osteoarthritis (OA)– and of pain experience on the interpersonal outcomes, *or* if there is (2) an interacting effect of diagnose and pain experience on the interpersonal outcomes. Our research questions were:

Does diagnose and/or the pain experience predict the expression of negative interpersonal interactions?

Does diagnose and/or the pain experience predict general level and type of interpersonal problems?

## Methods

### Participants

Participants were recruited at a specialized pain clinic, an orthopedic clinic, and at primary health care centers in the County Council of Region Sörmland between August 2016 and August 2020 (i.e., four years). Inclusion criteria for the case group was non-specific musculoskeletal pain (CMP) that had persisted for more than 6 months; age 18–65 years, mastery of the Swedish language, no self-reported substance abuse, and no serious medical or psychiatric condition (defined as continuous need of health care). Inclusion criteria for the control group was pain caused by osteoarthritis (OA), age 18–65 years, mastery of the Swedish language, no self-reported substance abuse, and no serious medical or psychiatric condition. Inclusion of male participants was controlled to match the case group.

### Interviewer and assessors of Core Conflictual Relationship Theme

The first author (PA) performed all research interviews with the participants. PA is a licensed psychologist and psychotherapist with 20 years of clinical experience from working with patients suffering from benign chronic pain. Authors BV and AL acted as assessors of interpersonal interactions. BV and AL are Ph.Ds., licensed psychologists, psychotherapists, researchers, and well acquainted with the CCRT method.

### Data

Patients’ narratives of interaction with others and self-rated interpersonal problems were defined as dependent variables, while Diagnose, Pain experience, and interaction between diagnose and pain experience (Diagnose*Pain experience) were defined as predictors. Age and work status were covariates that we controlled for.

### Core Conflictual Relationship Theme

Data on patients’ interaction with others was categorized with The Core Conflictual Relationship Theme-LU method (CCRT-LU; 37). CCRT-LU is a coding method applicable to interview transcripts. Patients’ descriptions of interactions with another person - relationship episodes - contain three components to be coded as: (a) a wish, need or intention (Wish); (b) the other person’s response (Response of Other); and (c) reaction to the others response (Response of Self). Each component is coded into one of two categories - harmonious or disharmonious, summarizing several sub-categories typical of each heading. Examples of harmonious sub-categories are being close, friendly, helpful, accepting, or self-confident. Examples of disharmonious sub-categories are being hostile, rejecting, irresponsible, or depressed.

Interviews to obtain material for the CCRT-LU coding followed a semi-structured protocol called the Relationship Anecdotes Paradigm [38]. Participants are asked for 10 relationship episodes involving any other person that took place anytime during the participant’s life. Relationship episodes not containing three components was later excluded from analysis. Thus, number of codes varies between participants.

For interrater reliability, 20% of the relationship episodes (67 out of 339 in total) were randomly selected and coded by the two judges BV and AL independently. Cohen’s Kappa [39] calculated on all three components was *k* = 0.72. Calculated for each component it was: *k*_wish_ = 0.46, *k*_response of other_ = 0.82 and *k*_response of self_ = 0.72. Judgements that diverged were discussed and a second blind test of interrater reliability was made based on 5 randomized interviews, containing 46 relationship episodes. Cohen’s Kappa calculated on all three components was *k* = 0.86 (*k*_wish_ = 0.56, *k*_response of other_ = 0.96 and *k*_response of self_ = 0.80). Low kappa value for the Wish component was caused by skewed distribution (high frequency of harmonious wishes). In the second interrater reliability test, the assessors agreed on 88% of the coding of wishes.

### General level and specific type of interpersonal problems

Inventory of Interpersonal Problems (IIP; 40), the 64-item circumplex version [41], is a self-report measure of a person’s most salient interpersonal problems. Items are rated on a 5-point Likert scale, ranging from zero (“not at all”) to four (“very much”). The mean of all items is interpreted as the general level of interpersonal distress, while eight subscales assess specific type of interpersonal problems. They are arranged along two axes of agency (dominant/submissive) and affiliation (cold/overly nurturant), and can be recalculated into four quadrants: Dominant/Intrusive, Cold/Vindictive, Socially avoidant/Nonassertive, and Exploitable/Overly nurturant [42]. The Swedish translation of IIP have adequate psychometric properties [43, 44].

### Predictors and covariates

Patients provided information on demographic and clinical variables, from which predictors and covariates were taken:

Pain experience is a composite index score based on four clinical variables about (1) pain severity, (2) time since onset, (3) number of health care visits related to pain during the last 12 months, (4) and pain being episodic or persistent. The score weights for the index were obtained through a principal component analysis (PCA) which showed an acceptable degree of shared variance between the four items for the analysis to be adequate (KMO = 0.67, Bartlett’s test of sphericity χ^2^ = 256.9, df = 6, *p* <.01). The underlying correlation matrix showed a positive association between pain severity and health care visits, as well as a negative association between these two variables and pain duration and pain intermittence. Thus, higher values on the index indicate more severe pain; shorter time since onset; more healthcare visits due to pain during the last 12 months; and pain being persistent rather than episodic. These variables are mandatory to pain assessments. Pain severity included in the index was measured with a subscale taken from The West Haven-Yale Multidimensional Pain Inventory (MPI; Kerns et al., 1985). The subscale has two items rated on a 7-point Likert-scale from zero (“no pain at all”) to six (“very intense”). The Swedish version of the instrument have adequate psychometric properties (r_test−retest_ = 0.75; internal consistency = 0.80; Bergström et al., 1998).

Work status is a composite score based on patient demographic information on whether they work at least 75% of full time or not, and whether they receive at least 75% of their income from sick-leave insurance or not. The variables were combined into a three-category variable: (1) working at least 75% and not receiving most of the income through sick-leave insurance; (2) working less than 75% and not receiving most of the income through sick-leave insurance; and (3) working less than 75% and receiving most of the income through sick-leave insurance).

### Statistical analyses

Initial checks of systematic differences between the case and control groups on relevant demographic and clinical variables were performed with t-tests for continuous data and *χ*^2^-test for nominal data. Data on IIP was positively skewed. No transformation of the IIP variables resulted in a sufficiently normal distribution; thus, these variables were categorized into an ordinal scale based on patients t-scores summarized over the two sub-scales included in each quadrant.

Generalized estimating equations (GEE) in SPSS version 28 was used to perform hierarchical binary regression analyses of the three CCRT components. The level 1 equation comprised the multiple, correlated measurements of CCRT components within everyone, whilst predictor variables and covariates were included at level 2. Predictor variables were Diagnose, Pain Experience, and Diagnose*Pain Experience. Age and Work Status were included as covariates. A binary log link function was assumed.

Multinomial logistic regression adopting a cumulative log link function through the GEE module in SPSS version 28 was used to analyze the ordinal dependent IIP-variables. Predictor variables were Diagnose, Pain Experience and Diagnose*Pain Experience. Age and Work status were included as covariates.

The decision to perform logistic regressions using the GEE framework rather than the standard logistic regression within the SPSS package was based on the ability to handle dependence due to clustering of data within individuals. Both the hierarchical binary regression and the multinomial logistic regression analyses results in partial regression coefficients. If the interaction variable Diagnose*Pain Experience is found to be significant, no conclusions of the main variables Diagnose and Pain Experience will be made.

### Procedure

Individuals suffering from CMP were recruited at a regional pain rehabilitation clinic to which they had been referred for multidisciplinary assessment. Diagnose of CMP was made by admitting physicians supported by physiotherapists. Patients were approached and given a written information about the study while making visits to the clinic. At the end of the information letter, a separate section for personal and contact information was answered and returned by interested patients. The control group was recruited through staff at primary health care centers and the orthopedic clinic. Diagnose of OA was made by physicians who had referred patients to specialized treatments. Potential participants were contacted by the first author. A short telephone interview was made to make sure they knew of no other cause of their pain than osteoarthritis. Participants were scheduled for the research interview and answering of questionnaires after consent was given.

The research visit took place either at the pain clinic or at a local primary health care center. The length of each visit varied between one to two hours, depending on the participant’s style in responding to the interview and the questionnaires. During the interview, each patient was asked to describe ten interactions with another person, one person for each episode. The interviewer was free to give prompts and ask follow-up questions until she thought there was enough material to code an episode. The RAP interviews were audio recorded and transcribed. The interviewer marked relevant aspects for the judges to focus on when coding the CCRT before the transcripts were submitted to them.

Questionnaires (the IIP, demographic data, and clinical characteristics) were answered in a paper and pen format after the interview and were collected by the interviewer. Any reflections the patient might have about the interview or questionnaires were welcome at this point.

### Ethics

This project was approved by the Swedish Ethical Review Authority (2015/602 − 31/5). All participants were given written and oral information about the project before giving oral consent to participate. Data was pseudonymized and stored on data servers only accessible by personnel within the County Council. The files were secured by additional firewalls. Only the first author has access to the files and the pseudonymization key. The judges were given transcripts of interviews with all identifying information removed.

Participants were informed about their rights to cancel at any point during the project without consequences to health care, and that support would be arranged in the case of unwanted reactions. At the end of each visit, participants were offered a moment to reflect upon the interview and the self-report. No adverse event occurred during the project.

## Results

Most of the participants in the case group were women and approximately the same proportion of female/male participants were obtained in the control group. Most participants were born in Sweden and had a degree from senior high school or college. Significant differences between the groups were found in age and clinical characteristics concerning pain experience and source of income. Participants suffering from OA were older than patients with CMP, and their pain experience was characterized by lower pain severity, longer time since onset, fewer health care visits, and pain being episodic rather than persistent. Their income was more often from work than from sick-leave insurance, working at least 75% of fulltime and receiving less than 50% of their income from social security insurance than patients suffering from CMP (Table 1).

**Table 1:** Demographic data and clinical characteristics of the participants in the case- and control group

	CMP *N* = 19	OA *N* = 16	Test Statistics (df)	*p*-value
*Demographic data*				
Age *M* (*SD*)	39.5 (9.7)	57.4 (3.9)	*t*(33) = -6.92	***< 0.001***
Women freq (*%*)	18 (94.7)	14 (87.5)	*χ*^*2*^(1) = 0.58	*0.58*
Country of birth freq (%)			*χ*^*2*^(1) = 1.56	*0.31*
Sweden	18 (94.7)	13 (81.2)		
Europe	0	1 (6.2)		
Other	1 (5.3)	2 (12.5)		
Education level freq (*%*)			*χ*^*2*^(1) = 1.57	*0.45*
Elementary	3 (15.8)	1 (6.2)		
Senior high school	10 (52.6)	7 (43.8)		
College/University	6 (31.6)	8 (50.0)		
Work status freq (*%*)			*χ*^*2*^(1) = 10.49	***0.001***
≥ 75*%*	5 (26.3)	13 (81.2)		
≤ 50*%*	14 (73.7)	3 (18.8)		
*Clinical characteristics*				
Pain intensity *M* (*SD*)	4.4 (0.90)	3.1 (1.03)	*t* (33) = 4.01	***< 0.001***
Pain duration, months *M (SD)*	32.8 (19.89)	140.3(87.5)	*t* (31) = -3.59	***0.001***
Pain intermittence freq (*%*)			*χ*^*2*^(1) = 6.43	***0.017***
Episodic, recurring	1 (5.3)	7 (43.8)		
Persistent, never absent	16 (84.2)	9 (56.2)		
No answer	2 (10.5)	-		
Health care visits* freq (*%*)			*χ*^*2*^(1) = 8.53	***0.003***
0–4	6 (31.6)	14 (87.5)		
>4	10 (52.6)	2 (12.5)		
No answer	2 (10.5)	-		

Note. CMP = Chronic Musculoskeletal Pain; OA = Osteoarthritis; df = degrees of freedom.

* Health care visits that has taken place the last 12 months due to the chronic pain problem.

In both groups, the CCRT component Wish was most often coded as harmonious, while the other two components were equally often coded as harmonious or disharmonious (Table 2). Interpersonal problems, both general level of distress and specific problems, were rated close to the norm (T score = 50) in both groups. However, ratings ranged with highest level of interpersonal distress two standard deviations above the norm mean (T score = 70; Table 2).

**Table 2:** Descriptive data of dependent variables

	CMP *N* = 19	OA *N* = 16
CCRT^a^ freq (*%*)		
Wish	168 (91)	143 (93)
Response from Other	81 (44)	83 (54)
Response of Self	100 (54)	83 (54)
IIP^b^ Mean (*SD*) *min-max*		
Total mean^c^	53 (8.4) 42–70	49 (8.6) 35–71
D-I^d^	53 (8.9) 40–77	48 (7.8) 40–62
C-V^e^	53 (8.0) 42–66	48 (8.7) 21–62
S-N^f^	49 (14.4) 0*–68	51 (10.1) 40–80
E-O^g^	53 (8.5) 38–68	47 (8.0) 36–66

^a^CCRT = Core Conflictual Relationship Theme, frequency and percentage of harmonious components in 185 (case) and 154 (control) relationship episodes, ^b^IIP = Inventory of Interpersonal Problems, t-scores, ^c^Total mean = general level of interpersonal distress, ^d^D-I = Dominant-Intrusive, ^e^C-V = Cold-Vindictive, ^f^S-N = Socially Avoidant-Nonassertive, ^g^E-O = Exploitable-Overly nurturant

* One patient has negated all questions in these two sub-scales. When this patient was removed, mean t-score of S-N was 52 (*SD* = 8.7) and *min-max* = 37–68

### Prediction of Core Conflictual Relationship Themes

The analysis of the CCRT component Wish did not result in any of the included predictors or covariates showing a significant effect (Diagnose: Wald *χ*^*2*^ [1] = 1.13, ns; Pain experience: Wald *χ*^*2*^ [1] = 0.11, ns; Diagnose*Pain experience: Wald *χ*^*2*^ [1] = 0.05, ns; Age: Wald *χ*^*2*^ [1] = 0.08, ns; Work status: Wald *χ*^*2*^ [2] = 3.90, ns).

The analysis of the CCRT component Response of Other showed a significant effect of Diagnose (Wald *χ*^*2*^ [1] = 4.34, *p* =.04). There was a near significant effect of Pain experience (Wald *χ*^*2*^ [1] = 3.32, *p* =.07) and of Diagnose*Pain experience (Wald *χ*^*2*^ [1] = 3.46, *p* =.06). None of the covariates had any significant effect (Age: Wald *χ*^*2*^ [1] = 1.13, ns; Work status: Wald *χ*^*2*^ [2] = 0.20, ns). The odds of CMP patients perceiving others’ responses as disharmonious was 6.45 times the odds of patients with OA, after controlling for the other four variables in the equation.

The analysis of the CCRT component Response of Self showed a significant effect of Diagnose (Wald *χ*^*2*^ [1] = 4.45, *p* =.04). None of the other predictor variables or covariates had any significant effect (Pain experience: Wald *χ*^*2*^ [1] = 0.70, ns; Diagnose*Pain experience: Wald *χ*^*2*^ [1] = 0.69, ns; Age: Wald *χ*^*2*^ [1] = 1.59, ns; Work status: Wald *χ*^*2*^ [2] = 0.25, ns). The odds of patients suffering from CMP perceiving their own responses as disharmonious was 3.32 times the odds of patients with OA, after controlling for the other four variables in the equation.

### Prediction of general and specific type of interpersonal problems

Diagnose*Pain experience was a significant predictor of general interpersonal distress in the CMP group (Wald *χ*^*2*^ [1] = 10.86, *p* <.01). The result show that in patients with CMP, higher levels of Pain experience were associated with higher levels of general interpersonal distress. This association was not found in the control group. Because the interaction variable (Diagnose*Pain experience) was significant, Pain experience was not interpreted although it was significant (Wald *χ*^*2*^ [1] = 11.32, *p* <.01). None of the other predictor variables or covariates were significant (Diagnose: Wald *χ*^*2*^ [1] = 0.25, ns; Age: Wald *χ*^*2*^ [1] = 0.38, ns; Work status: Wald *χ*^*2*^ [2] = 2.96, ns).

Diagnose*Pain experience was a significant predictor of problems within the Socially Avoidant-Nonassertive quadrant of IIP in the CMP group (Wald *χ*^*2*^ [1] = 5.77, *p* <.02). The result show that in patients with CMP, higher levels of Pain experience were associated with higher levels of Socially Avoidant-Nonassertive problems. This association was not found in the control group. Because the interaction variable (Diagnose*Pain experience) was significant, Pain experience was not interpreted although it was significant (Wald *χ*^*2*^ [1] = 5.67, *p* <.02). None of the other predictor variables or covariates were significant (Diagnose: Wald *χ*^*2*^ [1] = 1.43, ns; Age: Wald *χ*^*2*^ [1] = 0.48, ns; Work status: Wald *χ*^*2*^ [2] = 2.15, ns).

Diagnose*Pain experience was a significant predictor of problems within the Dominant-Intrusive quadrant of IIP in the CMP group (Wald *χ*^*2*^ [1] = 5.05, *p* =.02). The result show that in patients with CMP, lower levels of Pain experience were associated with higher levels of Dominant-Intrusive interpersonal problems. This association was not found in the control group. Because the interaction variable (Diagnose*Pain experience) was significant, the other predictors were not interpreted although they were significant (Diagnose: Wald *χ*^*2*^ [1] = 4.50, *p* =.03); Pain experience: Wald *χ*^*2*^ [1] = 4.93, *p* =.03). None of the covariates were significant (Age: Wald *χ*^*2*^ [1] = 0.99, ns; Work status: Wald *χ*^*2*^ [2] = 0.84, ns).

None of the predictor variables or covariates were significantly predicting problems within the Cold-Vindictive quadrant (Diagnose: Wald *χ*^*2*^ [1] = 1.90, ns; Pain experience: Wald *χ*^*2*^ [1] = 0.76, ns; Interaction Diagnose*Pain experience: Wald *χ*^*2*^ [1] = 0.73, ns; Age: Wald *χ*^*2*^ [1] = 2.95, ns; Work status: Wald *χ*^*2*^ [2] = 1.64, ns) or within the Exploitable-Overly Nurturant quadrant (Diagnose: Wald *χ*^*2*^ [1] = 0.43, ns; Pain experience: Wald *χ*^*2*^ [1] = 2.32, ns; Interaction diagnose*Pain experience: Wald *χ*^*2*^ [1] = 1.81, ns; Age: Wald *χ*^*2*^ [1] = 0.09, ns; Work status: Wald *χ*^*2*^ [2] = 0.65, ns) of IIP.

## Discussion

This study explored if interpersonal relationships differ depending on type of pain diagnose and pain experience. The Core Conflictual Telationship Theme coding of clinical interviews (CCRT) and the self-report Inventory of Interpersonal Problems (IIP) were used as interpersonal outcomes. Because pain experience, age, and work status significantly differed between diagnose groups, they were included as predictor and covariates. The main results were that in the interview, patients of either diagnose were equally likely to express harmonious needs or wishes towards others, but patients with chronic musculoskeletal pain (CMP) were significantly more likely to perceive the response of others a disharmonious, and to react with a disharmonious response to that. In the self-report, pain experience was significantly associated with interpersonal problems, but only in patients with CMP. Increased pain experience (i.e., more problematic) was significantly associated with elevation of interpersonal problems in general and with socially avoidant-nonassertive problems in particular. On the other hand, lower levels of pain experience were significantly associated with dominant-intrusive problems. No interaction effects of diagnose and pain experience on self-reported interpersonal problems were found in patients with OA.

The result that CMP patients were more likely to perceive others’ responses as disharmonious, despite a harmonious wish, and to express a disharmonious reaction to that themselves is similar to the result of a frustrated need for emotional support found in the study by Landa et al. [16]. Significantly more often than healthy controls did the patients in their study express interactions with others as a rejected desire for closeness, to which they reacted with anger, disappointment, or depression [16]. An important difference in our study is the comparison with a clinical group of patients and controlling for pain as a possible confounder. It could for example be argued that disharmonious interactions with others is a consequence of the aversive experience of pain. Instead, our results indicate that patients with CMP are more vulnerable to interpersonal issues than patients with OA. This is consistent with the notion that not all chronic pain conditions are equally affected by psychosocial factors [47], and a reminder that interpersonal relationships can be a crucial issue in patients whose chronic pain may be maintained by recurring interpersonal conflicts [19, 29].

In patients with CMP, self-reported interpersonal problems in general and socially avoidant-nonassertive problems in particular increased with the pain experience. A possible interpretation is that patients with CMP are more sensitive to others’ opinions, and that the need of others approval may become more important as the pain experience becomes more problematic. This interpretation is reasonable given a number of studies showing greater self-conscious emotions and doubt of others’ support in patients with non-specific pain (e.g., [47–50). Reversely, socially avoidant-nonassertive problems may cause more problems with pain. Borys et al. [23] could demonstrate that interpersonal vulnerability measured at baseline influenced the pain experience after an intervention. Chronic pain patients with concurrent problems of being too socially avoidant/nonassertive had significantly higher levels of pain, disability, depression, catastrophizing, and health care utilization following an intervention, compared to patients without such interpersonal problems at baseline. Thus, it seems that patients low on assertiveness run an increased risk of sub-optimal communication and treatments (e.g., [51]), which may contribute to the amplification of pain [27].

In patients with CMP, dominant-intrusive behaviours increased when the pain experience decreased. Dominance may impede social interaction, cooperation, and communication, and have been found to predict lower social support [21] and lower return to work after long term sick-leave [52]. However, our result can be interpreted as a sign of normal assertiveness in CMP patients whose subjective burden of pain is less problematic, exerting their agency to get the help they need. Although elevated, dominant/intrusive scores were within the normal range (Table 2). Thus, the inclination to be assertive decrease as the pain experience increase. Whether dominance is a problem or an asset to our participant is uncertain and could be a subject to further investigate.

The association between pain experience and interpersonal problems in patients with CMP, but not in patients with OA, indicates that type of pain diagnose matter. Faucett and Levine [33] found that in patients with arthritis, interpersonal conflict within the family was associated with less intense pain, while patients with chronic widespread pain (which is a type of CMP) reported the opposite: conflict within the family was associated with more pain. Patients with musculoskeletal pain were also more affected in their social networks (outside of the family) compared to patients with arthritis. We agree with their argument that when pain is consistent with organic findings, tasks and expectations are easier to negotiate. In the case of specific pain, such as arthritis, conflicts can be minimized in the face of an active relapse with the approval of others [33]. In contrast, patients with CMP will be more uncertain about getting their pain recognized as a pretext to withdraw from tasks and expectations.

This study contributes to a social perspective on chronic pain where interpersonal problems can be a distinct factor to address. Our results add to an established understanding of some chronic pain conditions as more affected by psychosocial factors than others (e.g., [53–55]), and to the suggested division of pain into primary and secondary pain [47]. In CMP, representing primary pain, the underlying pain mechanism is in the central nervous system where emotional distress - including interpersonal distress - contributes to the pain maintaining process [26–28]. In OA, representing secondary pain, the underlying mechanism is another disease, affecting bones, joints, or related soft tissue [47]. Others have already proposed that contemporary pain treatment packages with standardized content should gain from a more individualized approach [56, 57]. Supplementing pain management treatment with psychotherapy would represent such an individualization.

The strength of this study is the use of a control group. Because pain can impede interpersonal interactions, controlling for it becomes crucial. We also believe that the use of two types of measures, representing both clinicians’ and patients’ perspective, offers a wider picture of interpersonal issues in chronic pain. Another strength is the import of measures from psychiatric and psychotherapeutic settings. Despite the common understanding of chronic pain as a biopsychosocial phenomenon, measures of interpersonal functioning have been lacking, or have been restricted to a narrow definition of solicitous support in the instance of pain.

There are some major limitations of this study. First, the number of participants in each group were small, uneven, and unmatched, with significant differences in their pain experience, work status and age. Those variables had to be included in the analyses, resulting in an underpowered study. Although such differences are a real-world fact - patients with CMP are for example younger than patients with OA (e.g., [58,59]) we draw our conclusions with great care. It is for example possible that patients with OA have had more time to learn how to deal with pain more efficiently due to their higher average age and/or longer pain duration. Second, it is possible that a study about relationships attracts certain participants and not others, leading to a volunteer bias that limits the generalizability of the results.

Future research on the interpersonal dimension of CMP could gain from yet a third perspective if spouses or families were included in the measures. Complementing patients and expert assessment with them would result in a fuller picture of interpersonal behaviours as a problem or an asset.

## Electronic supplementary material

Below is the link to the electronic supplementary material.

Supplementary Material 1

Supplementary Material 2

## Data Availability

The data in this study can be considered personal and can not be shared openly, but will be made available upon reasonable request through the corresponding author.

## Abbreviations

CCRT: Core Conflictual Relationship Theme
CMP: Chronic Musculoskeletal Pain
IIP: Inventory of Interpersonal Problems
OA: Osteoarthritis
