# Supplementary material for: Interpersonal relationships in patients suffering from chronic musculoskeletal pain: a case-control study analyzing core conflictual relationship themes and interpersonal problems
Source: Biopsychosoc Med. 2025 Jul 28;19:14. doi: 10.1186/s13030-025-00335-x (PMC12302453; doi:10.1186/s13030-025-00335-x)
Supplement: Supplementary file 1 — Supplementary Material 1 [file 13030_2025_335_MOESM1_ESM.docx]

**Interpersonal relationships in patients suffering from chronic musculoskeletal pain: A case-control study analyzing core conflictual relationship themes and interpersonal problems**

Pernilla Abrahamsson^a*^, M.Sc, Bo Vinnars^b^, PhD, and Annika Lindgren^c^, PhD^.^

^a^ Division of Clinical Psychology, Department of Psychology, Box 1225 Uppsala University, 751 42 Uppsala, Sweden. ∗ Corresponding author. E-mail: [pernilla.abrahamsson@psyk.uu.se](mailto:pernilla.abrahamsson@psyk.uu.se)

^b^ Department of Psychology, Stockholm University, 106 91 Stockholm, Sweden.

^c^ Department of Clinical Neuroscience, Karolinska Institute, 171 77 Stockholm, Sweden.
