# Supplementary material for: Interpersonal relationships in patients suffering from chronic musculoskeletal pain: a case-control study analyzing core conflictual relationship themes and interpersonal problems
Source: Biopsychosoc Med. 2025 Jul 28;19:14. doi: 10.1186/s13030-025-00335-x (PMC12302453; doi:10.1186/s13030-025-00335-x)
Supplement: Supplementary file 2 — Supplementary Material 2 [file 13030_2025_335_MOESM2_ESM.docx]

**Abstract**

**Background**

Psychosocial factors are involved in all types of chronic pain but seem to play a more prominent role in non-specific pain, such as chronic musculoskeletal pain (CMP), compared to a specific pain condition, such as osteoarthritis (OA). We explored if diagnose and the pain experience in patients with CMP predicted more problematic interpersonal relationships compared to patients with OA.

**Material and methods**

Nineteen patients with CMP and 16 unmatched clinical controls with OA were measured with the Core Conflictual Relationship Theme coding of clinical interviews (CCRT) and the Inventory of Interpersonal Problems (IIP).

**Results**Significant differences in age, work status, and pain experience were found between the groups. Controlling for these variables, components of CCRT were significantly more likely to be disharmonious in patients with CMP compared to patients with OA. Patients with CMP also reported more interpersonal distress in general and socially avoidant-nonassertive problems in particular as their pain experience increased. Conversely, scores of dominant-intrusive behaviours increased as their pain experience decreased. These interaction effects between pain experience and interpersonal problems were not seen in patients with OA.

**Conclusions**

The impact of interpersonal issues may differ depending on type of pain diagnosis. This study show that interpersonal distress seems to play a more prominent role in non-specific chronic pain compared to a specific pain condition. It is possible that patients whose pain-processing system is burdened by interpersonal problems are more prone to non-specific pain, such as CMP. It could also be that primary pain is a greater challenge to interpersonal relationships. Whether interpersonal distress is a precursor or an additional stressor, it may worsen the condition of primary pain with implications for treatments.

**Keywords**
Chronic pain, Pain diagnosis, Pain experience, Interpersonal relationships, Interpersonal problems.
